# On Fractional Model Reference Adaptive Control

**DOI:** 10.1155/2014/521625

**Published:** 2014-01-16

**Authors:** Bao Shi, Jian Yuan, Chao Dong

**Affiliations:** Institute of System Science and Mathematics, Naval Aeronautical and Astronautical University, Yantai 264001, China

## Abstract

This paper extends the conventional Model Reference Adaptive Control systems to fractional ones based on the theory of fractional calculus. A control law and an incommensurate fractional adaptation law are designed for the fractional plant and the fractional reference model. The stability and tracking convergence are analyzed using the frequency distributed fractional integrator model and Lyapunov theory. Moreover, numerical simulations of both linear and nonlinear systems are performed to exhibit the viability and effectiveness of the proposed methodology.

## 1. Introduction

Fractional calculus dates back to the end of the 17th century. Over three hundred years, a firm theoretical foundation has been established due primarily to Liouville, Grünwald, Letnikov, Riemann, and Caputo. In the last three decades, many scientific studies have shown the importance of fractional calculus and its applications in mathematics, physics, chemistry, material, engineering, finance, and even social science [1–3]. The stability of fractional differential equations and fractional control has gained rapid development very recently [4–6].

Several pioneering attempts to develop fractional order control methodologies have been made, such as TID control [7], CRONE control [8], fractional PID control [9], and fractional lead-lag compensator [10]. Basic ideas and technical formulations of the above four fractional control schemes with comparative comments have been addressed in [11]. By applying fractional calculus to advanced nonlinear control theory, several fractional nonlinear control schemes have been proposed very recently, such as fractional sliding mode control [12–24], fractional adaptive control [25–29], and fractional optimal control [30–32]. Particularly, in [25], the authors have presented two ideas to extend the conventional Model Reference Adaptive Control (MRAC) by using fractional order parameter adjustment rule and fractional reference model. In [26], a fractional model reference adaptive control algorithm for SISO plants has been proposed in frequency domain, which guarantees the stability and ability to reject disturbances.

Inspired by contributions from [25, 26], this paper aims at going further by applying an incommensurate fractional adaptation law to fractional plant and fractional reference model. Furthermore, the stability and tracking convergence of the fractional adaptive system are analyzed based on the continuous frequency distributed model of fractional integrator.

The rest of the paper is organized as follows.  Section 2 reviews some basic definitions and theorems about fractional calculus.  Section 3 designs a control law and a fractional adaptation law for fractional linear MRAC systems along with numerical simulations.  Section 4 extends the proposed schemes to fractional nonlinear systems. Finally, Section 5 concludes this paper with some remarks on future study.

## 2. Basic Definitions and Preliminaries

Fractional calculus is a generalization of integration and differentiation to noninteger order fundamental operator _*a*_ *D*_*t*_^*α*^, where *a* and *t* are the bounds of the operation and *a* ∈ ℝ. The three most frequently used definitions for the general fractional calculus are the Grünwald-Letnikov definition, the Riemann-Liouville definition, and the Caputo definition [1–3].

**Definition 1**

The Grünwald-Letnikov derivative definition of order *α* is described as

(1) $$\begin{array}{c} {}_{a}D_{t}^{\alpha }f\left(t\right)=\underset{h\to 0}{\lim}\frac{1}{h^{\alpha }}\sum_{j=0}^{\infty }{\left(-1\right)}^{j}\left(\begin{array}{c} \alpha \\ j \end{array}\right)f\left(t-jh\right). \end{array}$$

**Definition 2**

The Riemann-Liouville derivative definition of order *α* is described as

(2) $$\begin{array}{c} {}_{a}D_{t}^{\alpha }f\left(t\right)=\frac{1}{\Gamma \left(n-\alpha \right)}\frac{d^{n}}{dt^{n}}\int_{a}^{t}\frac{f\left(\tau \right)d\tau }{{\left(t-\tau \right)}^{\alpha -n+1}}. \end{array}$$

**Definition 3**

The Caputo definition of fractional derivatives can be written as

(3) $$\begin{array}{c} {}_{a}D_{t}^{\alpha }f\left(t\right)=\frac{1}{\Gamma \left(n-\alpha \right)}\int_{a}^{t}\frac{f^{\left(n\right)}\left(\tau \right)d\tau }{{\left(t-\tau \right)}^{\alpha -n+1}},\;n-1<\alpha <n. \end{array}$$

In the following, we use the Caputo approach to describe fractional systems and the Grünwald-Letnikov approach to perform numerical simulations. To simplify the notation, we denote the fractional derivative of order *α* as *D* ^*α*^ instead of _0_ *D*_*t*_^*α*^ in this paper.

**Lemma 4 (the continuous frequency distributed model [33])**

The fractional integrator *D* ^−*α*^, 0 < *α* < 1 is a linear frequency distributed system, with input *v*(*t*) and output *x*(*t*). Its frequency distributed state *z*(*ω*, *t*) verifies the differential equation (for the elementary frequency *ω*) as follows:

(4) $$\begin{array}{c} \frac{\partial z\left(\omega ,t\right)}{\partial t}=-\omega z\left(\omega ,t\right)+v\left(t\right), \end{array}$$

and the output *x*(*t*) of the fractional integrator is the weighted integral (with weight *μ*(*ω*)) of all the contributions *z*(*ω*, *t*) ranging from 0 to *∞* as follows:

(5) $$\begin{array}{c} x\left(t\right)=\int_{0}^{\infty }\mu \left(\omega \right)z\left(\omega ,t\right)d\omega , \end{array}$$

with *μ*(*ω*) = (sin(*απ*)/*π*)*ω* ^−*α*^.

The relations (4) and (5) define the frequency distributed model of the fractional integrator.

**Lemma 5**

The quadratic form *W* = *W*_1_ + *W*_2_ is positive semidefinite if *a*_*i*_ ≥ 0, where *W*_1_ = ∑_*i*=1_^*m*^ *W*_1*i*_ and *W*_2_ = ∑_*i*=1_^*m*^ *a*_*i*_ *W*_2*i*_ with *W*_1*i*_ = ∫_0_^*∞*^ *μ*_*i*_(*ω*)*ωz*_*i*_^2^ *dω* and *W*_2*i*_ = *x*_*i*_^2^, *i* = 1,2,…, *m* [34].

## 3. Adaptive Control of Fractional Linear Systems

In this section, we extend the conventional MRAC systems to fractional ones based on the theory of fractional calculus. Firstly, a fractional plant and an incommensurate fractional reference model are described by the fractional differential equations. Then, a control law and an incommensurate fractional adaptation law which are generalized from the conventional ones [35, 36] are designed. Finally, the stability and tracking performance of the fractional adaptive system are analyzed based on the continuous frequency distributed model of fractional integrator.

### 3.1. Fractional Adaptive Control Design

Consider the following fractional differential equation:

(6) $$\begin{array}{c} D^{\alpha_{1}}y=-a_{p}y+b_{p}u, \end{array}$$

where *α*_1_ is the fractional order lying between (0,1), *y* is the plant output, *u* is the input, and *a*_*p*_ and *b*_*p*_ are constant plant parameters that are assumed to be unknown.

The reference model is specified by a fractional differential equation as follows:

(7) $$\begin{array}{c} D^{\alpha_{1}}y_{m}=-a_{m}y_{m}+b_{m}r\left(t\right), \end{array}$$

where *a*_*m*_ and *b*_*m*_ are constant parameters and *r*(*t*) is a bounded external reference signal.

Our objective of the fractional adaptive control design is to construct a control law and a fractional adaptation law to make the fractional plant (6) track the fractional reference model (7) on the basis of system stability.

Let us assume the sign of the parameter *b*_*p*_ to be known and design the control law to be

(8) $$\begin{array}{c} u={\hat{a}}_{r}\left(t\right)r+{\hat{a}}_{y}\left(t\right)y, \end{array}$$

where ${\hat{a}}_{r}\left(t\right)$ and ${\hat{a}}_{y}\left(t\right)$ are variable feedback gains to be decided later.

Define the tracking error

(9) $$\begin{array}{c} e=y-y_{m}, \end{array}$$

and the parameter errors

(10) $$\begin{array}{c} {\tilde{a}}_{r}={\hat{a}}_{r}-a_{r}^{*},\\ {\tilde{a}}_{y}={\hat{a}}_{y}-a_{y}^{*}, \end{array}$$

where *a*_r_* = *b*_*m*_/*b*_*p*_ and *a*_y_* = (*a*_*p*_ − *a*_*m*_)/*b*_*p*_ are the same as the conventional case.

Subtracting (7) from (6) derives the dynamics of tracking error as follows:

(11) $$\begin{array}{c} D^{\alpha_{1}}e=-a_{m}e+b_{p}\left({\tilde{a}}_{r}r+{\tilde{a}}_{y}y\right). \end{array}$$

To adjust the two parameters in the control law (8), an adaptation law can be chosen in the fractional form as follows

(12) $$\begin{array}{c} D^{\alpha_{2}}{\hat{a}}_{r}=-\mathrm{sign}\left(b_{p}\right)\gamma er,\\ D^{\alpha_{3}}{\hat{a}}_{y}=-\mathrm{sign}\left(b_{p}\right)\gamma ey, \end{array}$$

where 0 < *α*_2_ < 1, 0 < *α*_3_ < 1.

Note that the control law (8) and the fractional adaptation law (12) are generalized from the conventional MRAC systems [35, 36].

### 3.2. Analysis of Stability and Tracking Convergence

In the following, we will prove that the fractional plant (6) can be controlled with the control law (8) and the adaptation law (12).

The tracking error system (11) and adaptation law (12) constitute the following closed-loop adaptive system:

(13) $$\begin{array}{c} D^{\alpha_{1}}e=-a_{m}e+b_{p}\left({\tilde{a}}_{r}r+{\tilde{a}}_{y}y\right),\\ D^{\alpha_{2}}{\hat{a}}_{r}=-\mathrm{sign}\left(b_{p}\right)\gamma er,\\ D^{\alpha_{3}}{\hat{a}}_{y}=-\mathrm{sign}\left(b_{p}\right)\gamma ey. \end{array}$$

Based on the continuous frequency distributed model of the fractional integrator in Lemma 4, the above adaptive system is exactly equivalent to the infinite dimensional ODEs as follows:

(14) $$\begin{array}{c} \frac{\partial z_{1}\left(\omega ,t\right)}{\partial t}=-\omega z_{1}\left(\omega ,t\right)-a_{m}e+b_{p}\left({\tilde{a}}_{r}r+{\tilde{a}}_{y}y\right),\\ e\left(t\right)=\int_{0}^{\infty }\mu_{1}\left(\omega \right)z_{1}\left(\omega ,t\right)d\omega ,\\ \frac{\partial z_{2}\left(\omega ,t\right)}{\partial t}=-\omega z_{2}\left(\omega ,t\right)--\mathrm{sign}\left(b_{r}\right)\gamma er,\\ {\tilde{a}}_{r}\left(t\right)=\int_{0}^{\infty }\mu_{2}\left(\omega \right)z_{2}\left(\omega ,t\right)d\omega ,\\ \frac{\partial z_{3}\left(\omega ,t\right)}{\partial t}=-\omega z_{3}\left(\omega ,t\right)--\mathrm{sign}\left(b_{y}\right)\gamma ey,\\ {\tilde{a}}_{y}\left(t\right)=\int_{0}^{\infty }\mu_{3}\left(\omega \right)z_{3}\left(\omega ,t\right)d\omega , \end{array}$$

with *μ*_*i*_(*ω*) = (sin(*α*_*i*_ *π*)/*π*)*ω* ^−*α*_*i*_^, *i* = 1,2, 3.

In the above continuous frequency distributed model, *z*_1_(*ω*, *t*), *z*_2_(*ω*, *t*), and *z*_3_(*ω*, *t*) are the true state variables, while *e*(*t*), ${\tilde{a}}_{r}\left(t\right)$, and ${\tilde{a}}_{y}\left(t\right)$ are the pseudo state variables.

Let us define two types of Lyapunov functions as follows:

- *v*_*i*_(*w*, *t*): the monochromatic Lyapunov functions corresponding to the elementary frequency;
- *V*_*i*_(*t*): the Lyapunov functions summing all the monochromatic *v*_*i*_(*w*, *t*) with the weighting functions *μ*_*i*_(*ω*), *i* = 1,2, 3.

Namely,

(15) $$\begin{array}{c} v_{1}\left(w,t\right)=\frac{1}{2}z_{1}^{2},\\ v_{2}\left(w,t\right)=\frac{\left|b_{p}\right|}{2\gamma }z_{2}^{2},\\ v_{3}\left(w,t\right)=\frac{\left|b_{p}\right|}{2\gamma }z_{3}^{2},\\ V_{i}\left(t\right)=\int_{0}^{\infty }\mu_{i}\left(\omega \right)v_{i}\left(\omega ,t\right)d\omega ,\;i=1,2,3. \end{array}$$

Then,

(16) $$\begin{array}{c} \frac{dV_{1}}{dt}=\int_{0}^{\infty }\mu_{1}\left(\omega \right)\frac{\partial v_{1}\left(\omega ,t\right)}{\partial t}d\omega . \end{array}$$

Substituting the first equation of (14) into (16) gives

(17) dV1dt=∫0∞μ1(ω)z1[−ωz1(ω,t)       −ame+bp(a~rr+a~yy)]dω=−∫0∞μ1(ω)ωz12dω  +∫0∞μ1(ω)z1[−ame+bp(a~rr+a~yy)]dω=−∫0∞μ1(ω)ωz12dω+[−ame+bp(a~rr+a~yy)]  ×∫0∞μ1(ω)z1dω.

Substituting the second equation of (14) into the integral term of (17) yields

(18) $$\begin{array}{c} \frac{dV_{1}}{dt}=-\int_{0}^{\infty }\mu_{1}\left(\omega \right)\omega z_{1}^{2}d\omega -a_{m}e^{2}+eb_{p}\left({\tilde{a}}_{r}r+{\tilde{a}}_{y}y\right). \end{array}$$

Similarly, one derives

(19) $$\begin{array}{c} \frac{dV_{2}}{dt}=-\frac{\left|b_{p}\right|}{\gamma }\int_{0}^{\infty }\mu_{2}\left(\omega \right)\omega z_{2}^{2}d\omega -eb_{p}{\tilde{a}}_{r}r,\\ \frac{dV_{3}}{dt}=-\frac{\left|b_{p}\right|}{\gamma }\int_{0}^{\infty }\mu_{3}\left(\omega \right)\omega z_{3}^{2}d\omega -eb_{p}{\tilde{a}}_{y}y. \end{array}$$

Finally, lets define

(20) $$\begin{array}{c} V\left(t\right)=V_{1}\left(t\right)+V_{2}\left(t\right)+V_{3}\left(t\right). \end{array}$$

Then, one derives

(21) dVdt=−∫0∞μ1(ω)ωz12dω−|bp|γ∫0∞μ2(ω)ωz22dω−|bp|γ∫0∞μ3(ω)ωz32dω−ame2.

Owing to Lemma 5, *dV*/*dt* is negative semidefinite, implying the stability of the fractional adaptive system (13).

This proves that the fractional MRAC problem (6)-(7) can be solved by using the control law (8) and the fractional adaptation law (12).

### 3.3. Numerical Simulations

Consider the control of the fractional plant with known fractional order *α*_1_ = 0.9 and unknown parameters *a*_*p*_ and *b*_*p*_. The sign of *b*_*p*_ is assumed to be positive. The fractional reference model is chosen to be

(22) $$\begin{array}{c} D^{0.9}y_{m}=-4y_{m}+4\sin\left(3t\right), \end{array}$$

that is, *α*_1_ = 0.9, *a*_*m*_ = 4, *b*_*m*_ = 4, *r*(*t*) = sin(3*t*).

The adaptation gain is chosen to be *γ* = 1, while the fractional orders of the adaptation law are chosen as *α*_2_ = 0.4, *α*_3_ = 0.4.

As for the initialization issue, we refer to the method proposed by Lorenzo and Hartley in [37], where it is addressed that the initial conditions for fractional differential equations with order between 0 and 1 are a constant function of time. Therefore, the initial conditions of the fractional plant, the fractional model, and the fractional adaptation law are chosen, respectively, as *y*(*t*) = *y*(0^+^) = 10, *y*_*m*_(*t*) = *y*_*m*_(0^+^) = 10, ${\hat{a}}_{r}\left(t\right)={\hat{a}}_{r}\left(0^{+}\right)=1$, ${\hat{a}}_{y}\left(t\right)={\hat{a}}_{y}\left(0^{+}\right)=1$, for −*∞* ≤ *t* ≤ 0.

**Figure 1:**
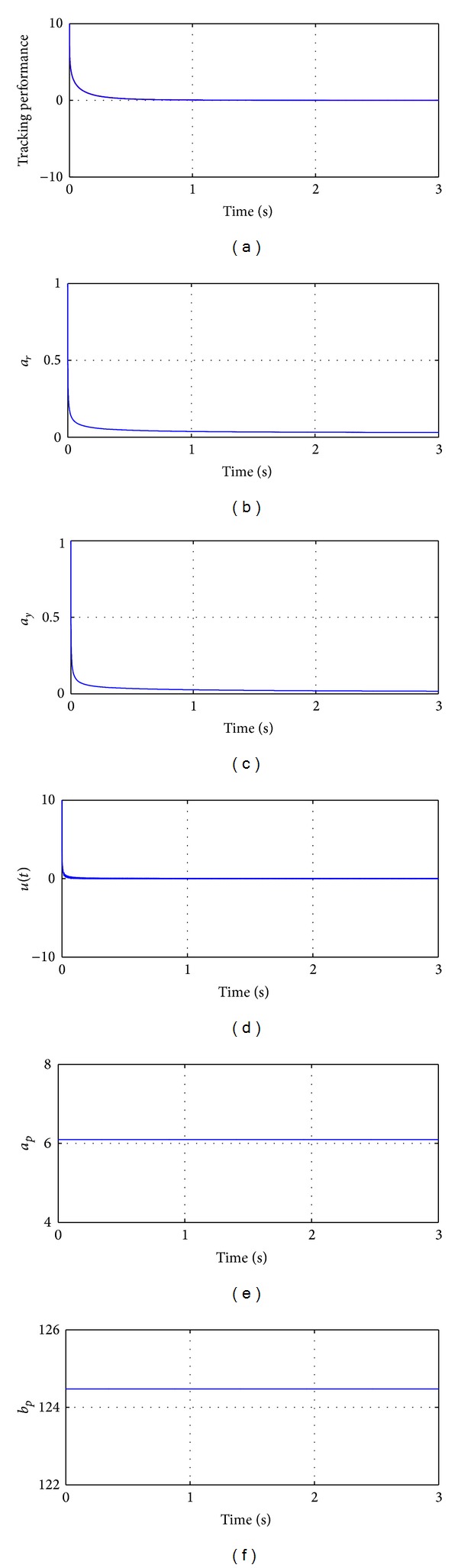
Fractional adaptive control of the fractional linear system (6). (a) Tracking performance (red line represents the state of the fractional plant, while the blue line represents the state of the fractional reference model); (b) control parameter ${\hat{a}}_{r}\left(t\right)$; (c) control parameter ${\hat{a}}_{y}\left(t\right)$; (d) control input *u*(*t*); (e) estimation of parameter *a*_*p*_(*t*); (f) estimation of parameter *a*_*y*_(*t*).

The numerical simulations of the behavior of the fractional linear adaptive system are illustrated in Figure 1. For interpretations of the references to the color in the upper left figure, the reader is referred to the web version of this paper.

**Remark 6**

In [25], the authors have designed a commensurate fractional adaptation law for the integer order SISO systems. Benefits from the use of fractional calculus are also illustrated mainly via numerical simulations. However, detailed theoretical analysis is left out in their work. In the following, we give the theoretical analysis of the fractional control for the integer order plant.

With the first order error dynamics (i.e., *α*_1_ = 1 in system (13)) and the fractional adaptation law (12), the closed-loop adaptive system is described by

(23) $$\begin{array}{c} \frac{de}{dt}=-a_{m}e+b_{p}\left({\tilde{a}}_{r}r+{\tilde{a}}_{y}y\right),\\ D^{\alpha_{2}}{\hat{a}}_{r}=-\mathrm{sign}\left(b_{p}\right)\gamma er,\\ D^{\alpha_{3}}{\hat{a}}_{y}=-\mathrm{sign}\left(b_{p}\right)\gamma ey. \end{array}$$

By converting the last two FDEs into infinite-dimensional ODEs as (14) and introducing Lyapunov function as

(24) $$\begin{array}{c} V\left(t\right)=\frac{1}{2}e^{2}+\frac{\left|b_{p}\right|}{2\gamma }\int_{0}^{\infty }\mu_{2}\left(\omega \right)z_{2}^{2}d\omega +\frac{\left|b_{p}\right|}{2\gamma }\int_{0}^{\infty }\mu_{3}\left(\omega \right)z_{3}^{2}d\omega , \end{array}$$

one derives

(25) dVdt(t)=−|bp|γ∫0∞μ2(ω)ωz22dω−|bp|γ∫0∞μ3(ω)ωz32dω−ame2.

By Lemma 5, *dV*/*dt* is negative semidefinite, which implies the stability of the fractional adaptive system (23). This proves that the integer order plant (*α*_1_ = 1 in system (6)) can be controlled with the control law (8) and the fractional adaptation law (12).

**Remark 7**

From the analysis in Remark 6, it is evident that the values of the fractional orders *α*_1_, *α*_2_, and *α*_3_ can be extended to the following values: 0 < *α*_1_ ≤ 1, 0 < *α*_2_ ≤ 1, and 0 < *α*_3_ ≤ 1.

## 4. Extension to Fractional Nonlinear Systems

In this section, we extend the fractional control method previously proposed to fractional nonlinear systems. The fractional nonlinear plant is described by the fractional differential equation as follows:

(26) $$\begin{array}{c} D^{\alpha_{1}}y=-a_{p}y-c_{p}f\left(y\right)+b_{p}u, \end{array}$$

where *f* is a known nonlinear function. The fractional reference model is chosen as (7). Instead of using control law (8) and adaptation law (12), we now use the following control law:

(27) $$\begin{array}{c} u={\hat{a}}_{r}\left(t\right)r+{\hat{a}}_{y}\left(t\right)y+{\hat{a}}_{f}\left(t\right)f\left(y\right), \end{array}$$

and the adaptation law

(28) $$\begin{array}{c} D^{\alpha_{2}}{\tilde{a}}_{r}=-\mathrm{sign}\left(b_{p}\right)\gamma er,\\ D^{\alpha_{3}}{\tilde{a}}_{y}=-\mathrm{sign}\left(b_{p}\right)\gamma ey,\\ D^{\alpha_{4}}{\tilde{a}}_{f}=-\mathrm{sign}\left(b_{p}\right)\gamma ef\left(y\right), \end{array}$$

with ${\tilde{a}}_{f}={\hat{a}}_{f}-c_{p}/b_{p}$.

Similarly, one can easily analyze the stability and tracking convergence of the above fractional nonlinear adaptive system based on the continuous frequency distributed model of fractional integrator.

The following example demonstrates the behavior of the fractional nonlinear adaptive system.

Consider the fractional nonlinear plant with known fractional order *α*_1_ = 0.9 and unknown parameters *a*_*p*_, *b*_*p*_, and *c*_*p*_. The sign of *b*_*p*_ is assumed to be positive. *f*(*y*) is chosen to be *y* ^2^. The fractional reference model is chosen to be the same as (22).

The initial conditions of the fractional plant, the fractional model, and the fractional adaptation law are chosen, respectively, as *y*(*t*) = *y*(0^+^) = 10, *y*_*m*_(*t*) = *y*_*m*_(0^+^) = 10, ${\hat{a}}_{r}\left(t\right)={\hat{a}}_{r}\left(0^{+}\right)=1$, ${\hat{a}}_{y}\left(t\right)={\hat{a}}_{y}\left(0^{+}\right)=1$, ${\hat{a}}_{f}\left(t\right)={\hat{a}}_{f}\left(0^{+}\right)=1$, for −*∞* ≤ *t* ≤ 0.

The adaptation gain is chosen to be *γ* = 1, while the fractional orders of the adaptation law are chosen as *α*_2_ = 0.9, *α*_3_ = 0.6, *α*_4_ = 0.6.

**Figure 2:**
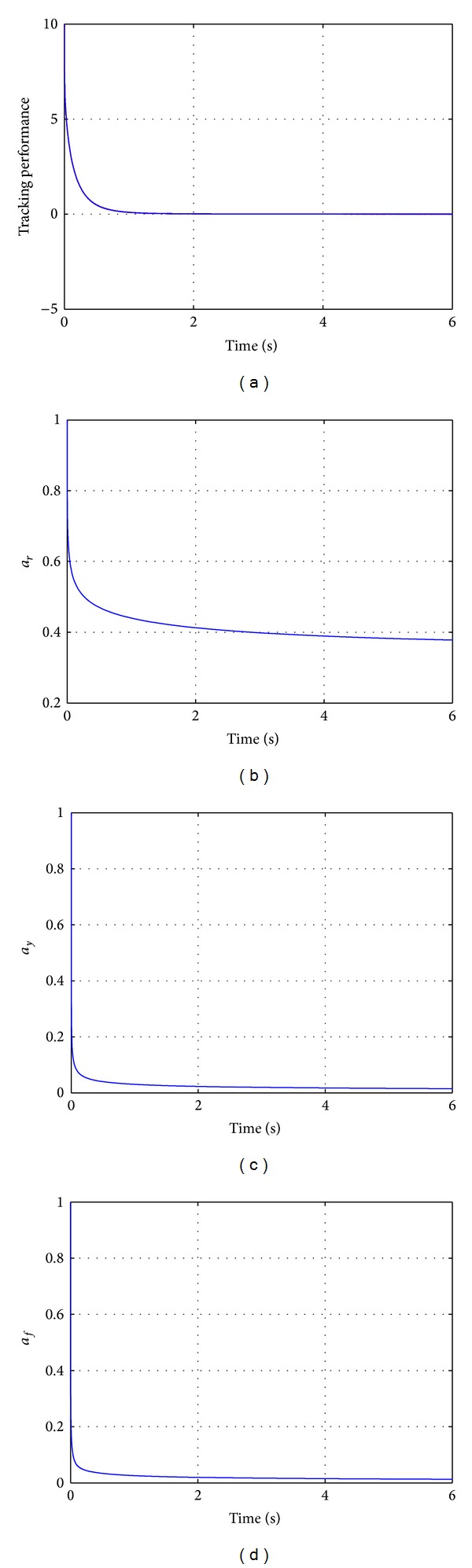
Fractional adaptive control of the fractional nonlinear system (26). (a) Tracking performance (red line represents the state of the fractional nonlinear plant, while, blue line represents the state of the fractional reference model); (b) control parameter ${\hat{a}}_{r}\left(t\right)$; (c) control parameter ${\hat{a}}_{y}\left(t\right)$; (d) control parameter ${\hat{a}}_{f}\left(t\right)$.

**Figure 3:**
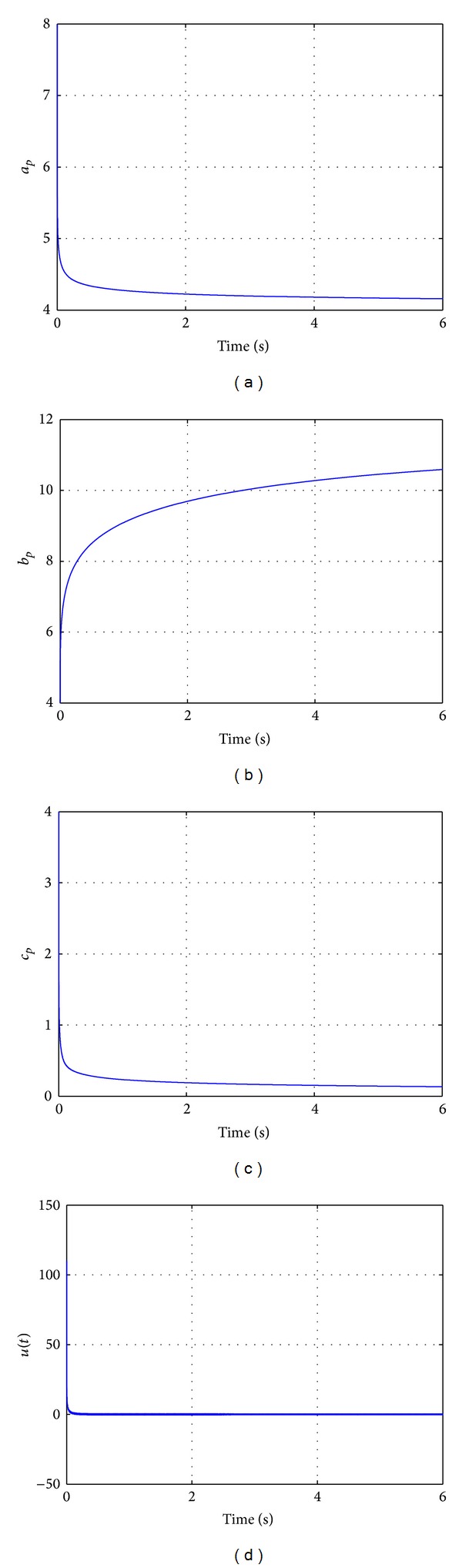
Fractional adaptive control of the fractional nonlinear system (26). (a) Estimation of parameter *a*_*p*_(*t*); (b) parameter *b*_*p*_(*t*); (c) estimation of parameter *c*_*p*_(*t*); (d) control input *u*(*t*).

Figures 2 and 3 illustrate the numerical simulations of the behavior of the fractional nonlinear adaptive system. For interpretations of the references to the color in the upper left figure of Figure 2, the reader is referred to the web version of this paper.

## 5. Concluding Remarks

Based on the theory of fractional calculus, this paper has extended the conventional MRAC systems to fractional ones by designing a control law and a fractional adaptation law for the fractional plant and fractional reference model. The stability and tracking convergence have been analyzed using the frequency distributed fractional integrator model and Lyapunov theory. Moreover, numerical simulations of both linear and nonlinear systems have been performed to exhibit the viability and effectiveness of the proposed methodology.

As for the future perspectives, our research efforts will be focused on the following.

- How the fractional orders of the adaptation law affect the performance of the control system.
- The optimal design of the fractional orders of the adaptation law.
- Superiority of fractional MRAC systems compared to the conventional ones.
- Design of fractional MRAC for general integer order or fractional order linear system, namely, *D* ^*α*^ *x* = *Ax* + *Bu*, 0 < *α* ≤ 1.
